# Role of exogenous abscisic acid in freezing tolerance of mangrove *Kandelia obovata* under natural frost condition at near 32^°^N

**DOI:** 10.1186/s12870-022-03990-2

**Published:** 2022-12-19

**Authors:** Xing Liu, Xiang Lu, Sheng Yang, Yu Liu, Wenqing Wang, Xin Wei, Hongjiu Ji, Bo Zhang, Wenzhen Xin, Junxiu Wen, Jinwang Wang, Qiuxia Chen

**Affiliations:** 1 Wenzhou Key Laboratory of Resource Plant Innovation and Utilization, Zhejiang Institute of Subtropical Crops, Zhejiang Academy of Agricultural Sciences, Wenzhou, 325005 Zhejiang China; 2 Key Laboratory of the Ministry of Education for Coastal and Wetland Ecosystems, College of the Environment and Ecology, Xiamen University, Xiamen, 361102 Fujian China; 3 Marine Fisheries Research Institute of Jiangsu Province, Nantong, 226007 Jiangsu China; 4 Wenzhou Municipal Key Laboratory for Applied Biomedical and Biopharmaceutical Informatics, Wenzhou-Kean University, Wenzhou, 325060 Zhejiang China; 5 College of Life and Environmental Sciences, Wenzhou University, Wenzhou, 325000 Zhejiang China

**Keywords:** *Kandelia obovata*, Cold stress, Abscisic acid, Physiological responses, Transcriptomic analysis, Phenylpropanoid metabolism

## Abstract

**Background:**

Mangroves possess substantial ecological, social, and economic functions in tropical and subtropical coastal wetlands. *Kandelia obovata* is the most cold-resistance species among mangrove plants, with a widespread distribution in China that ranges from Sanya (18° 12′ N) to Wenzhou (28° 20′ N). Here, we explored the temporal variations in physiological status and transcriptome profiling of *K. obovata* under natural frost conditions at ~ 32^o^N, as well as the positive role of exogenous abscisic acid (ABA) in cold resistance.

**Results:**

The soluble sugar (SS) and proline (Pro) functioned under freezing stress, of which SS was more important for *K. obovata*. Consistently, up-regulated DEGs responding to low temperature were significantly annotated to glycometabolism, such as starch and sucrose metabolism and amino sugar and nucleotide sugar metabolism. Notably, the top 2 pathways of KEGG enrichment were phenylpropanoid biosynthesis and flavonoid biosynthesis. For the antioxidant system, POD in conjunction with CAT removed hydrogen peroxide, and CAT appeared to be more important. The up-regulated DEGs responding to low temperature and ABA were also found to be enriched in arginine and proline metabolism, starch and sucrose metabolism, and peroxisome. Moreover, ABA triggered the expression of *P5CS* and *P5CR*, but inhibited the *ProDH* expression, which might contribute to Pro accumulation. Interestingly, there was no significant change in malondialdehyde (MDA) content during the cold event (*P* > 0.05), suggesting foliar application of ABA effectively alleviated the adverse effects of freezing stress on *K. obovata* by activating the antioxidant enzyme activity and increasing osmolytes accumulation, such as Pro, and the outcome was proportional to ABA concentration.

**Conclusions:**

This study deepened our understanding of the physiological characters and molecular mechanisms underlying the response of *K. obovata* to natural frost conditions and exogenous ABA at the field level, which could provide a sound theoretical foundation for expanding mangroves plantations in higher latitudes, as well as the development coastal landscape.

**Supplementary Information:**

The online version contains supplementary material available at 10.1186/s12870-022-03990-2.

## Background

Mangroves are the only woody halophytes living at the confluence of land and sea along tropical and subtropical tidal wetlands [1, 2]. They possess substantial ecological and commercial functions, being an important habit for birds, fish, crustaceans, and shellfish; offering protection of coastal communities from storm surges, typhoons, and tsunamis, and being an accumulation site for sediment, carbon, and nutrients [2]. It is well known that mangroves are delimited in latitudinal range by varying sensitivity to cold, and natural distribution in China is from Sanya (18°12΄ N), Hainan Province, to Fuding (27°20΄ N), Fujian Province [3]. The Intergovernmental Panel on Climate Change predicted that the global mean temperature will increase by 1.5 ℃ or more over the next 20 years. If the temperature rises by more than 4.0 ℃, mangroves would extend to Nanjing (32°37΄ N), Jiangsu Province [4]. From 1980 to 2009, the average temperature in the southern coastal area of China increased by 0.5 ℃ every 10 years, of which the magnitude showed a gradually increasing trend with latitude (http://www.cma.gov.cn). Consequently, some attempts have been made to transplant mangroves in higher latitude, such as Wenzhou (27°56′ N), Taizhou (28°41΄ N), Zhoushan (29°93΄ N), and Shanghai (30°53΄ N) [4–7]. Global warming facilitates the range extensions of mangroves to higher latitude, whereas the accompanying climate instability, such as extreme cold event could lead to physiological damage, mortality, and/or range contraction [8, 9]. In January 2016, a successive historic freezing event (minimum -5.5 °C) occurred in Yueqing Bay (28°20′ N), Wenzhou, which caused severe damage or even complete loss of introduced mangroves. Therefore, it is of great significance to characterize and understand how mangroves adapt and acclimate to freezing temperature at higher latitude [4, 6, 9].

*Kandelia obovata,* a member of the genus *Kandelia* in the family Rhizophoraceae, is the most cold-tolerant mangrove species [2, 9]. Moreover, due to its specific viviparous phenomena, beautiful shape, and unique floral pattern, *K. obovata* is an excellent coastal wetland landscape tree [1, 2]. Low temperature limits nutrient intake [10], disturbs normal photosynthesis [7], accelerates reactive oxygen species (ROS) formation [11], and ultimately leads to cell membrane damage, metabolism disorder, and even causes death of *K. obovata* [6, 12, 13]. Abscisic acid (ABA) is a vital phytohormone triggering adaptive responses to enable plants to cope with unfavorable environments [14]. Consistently, cold stress is always accompanied by elevated endogenous ABA content in plants [15]. Many reports also showed that external application of ABA effectively improved cold tolerance in many plant species which include chickpea [16], pepper [17], wheat [18], sugarcane [19], bermudagrass [20], and grapevine [21]. However, the negative effect of ABA on chilling resistance was observed in mandarin [22]. To date, no study had been conducted on the role of exogenous ABA in *K. obovata* under low-temperature stress.

RNA deep-sequencing (RNA-seq) has been used in molecular studies of the environmental stress responses of plants [23], whereas previous studies on the responses of *K. obovata* to cold stress are mostly limited to the physiological level [4, 6, 7, 9–11, 13]. Moreover, the aforementioned studies on the cold stress of *K. obovata* focused on low temperatures above 0 ℃, primarily investigated the cooling treatment. The responses of *K. obovata* to natural freezing temperature remain largely unknown as extreme low-temperature events are relatively uncommon in mangroves distribution area and difficult to fully replicate via manipulative experiments [9]. In 2019, *K. obovata* seedlings were firstly introduced to Qigong, Jiangsu Province at 31^o^59′ N from Wenzhou (northern boundary of artificial cultivation), Zhejiang Province at 27^o^56′ N. The objective of this study was to explore the temporal variations in physiological status and transcriptome profiling of mangrove *K. obovata* under natural frost conditions, as well as the acquisition of cold-resistance capability after exogenous ABA application. This study provides some insights into the possible physiological and molecular mechanisms underlying *K. obovata* response to freezing stress at the field level. It is anticipated that this information will provide a sound theoretical foundation for expanding mangroves plantations in higher latitudes, as well as the development coastal landscape.

## Results

### Effect of exogenous ABA on osmolytes in *K. obovata* during natural frost conditions

**Fig. 1:**
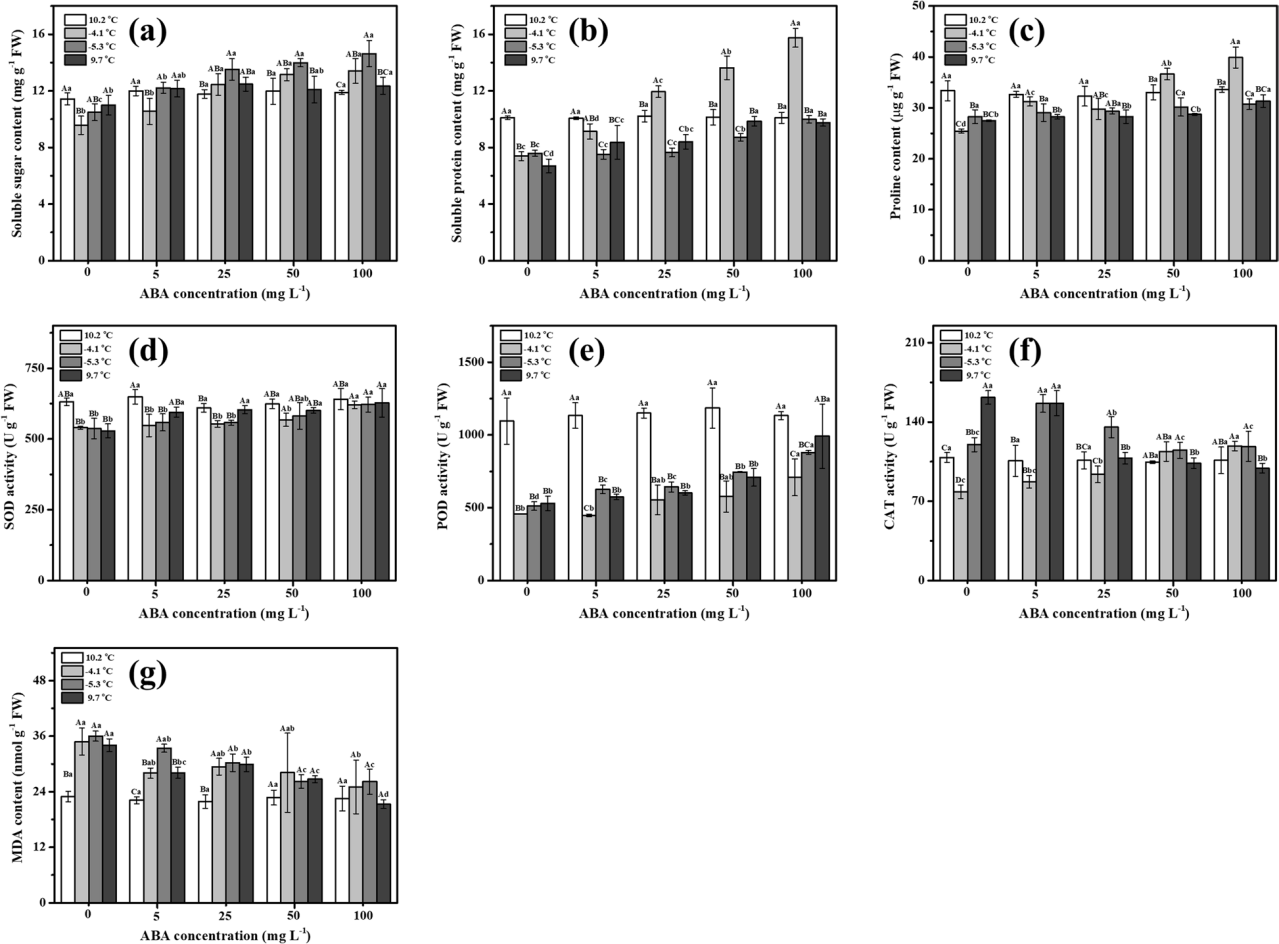
The temporal variations in physiological status of *K. obovata* under the natural frost conditions and exogenous ABA treatment. **a** soluble sugar content, **b** soluble protein content, **c** proline content, **d** SOD activity, **e** POD activity, **f** CAT activity, **g** MDA content. The data are presented as the means of three replicates (± SDs). Different uppercase letters indicate significant difference among different stages of cold event under the same ABA concentration at *P* < 0.05; lowercase letters indicate significant difference among different ABA concentrations under the same stage of cold event at *P* < 0.05

To study the relationships between physiological status and cold tolerance in *K. obovata*, it is imperative to study the varieties of osmotic adjustment substances during the cold event [24–27]. As shown in Fig. 1, cold stress dramatically reduced the osmolytes of *K. obovata* (*P* < 0.05) with contents of soluble sugar (SS, Fig. 1a), soluble protein (SP, Fig. 1b), and proline (Pro, Fig. 1c) decreasing from 11.4 mg g^−1^, 9.8 mg g^−1^, 33.4 µg g^−1^ to 9.6 mg g^−1^, 7.4 mg g^−1^, 25.4 µg g^−1^, respectively. Then the SS and Pro gradually increased with progress in persistence of low temperature. Notably, only SS returned to the original value before the cold event when temperature recovered (11.4 ± 0.4 mg g^−1^ *vs* 11.0 ± 0.7 mg g^−1^, *P* > 0.05), implying *K. obovata* suffered frost damage during the cold event. After ABA spraying, the osmolytes of *K. obovata* were significantly higher than control when initially subjected to cold stress (*P* < 0.05). For example, the SS, SP, and Pro of *K. obovata* treated with 100 mg L^−1^ ABA remarkably increased from 11.9 mg g^−1^, 10.1 mg g^−1^, and 33.6 µg g^−1^ to 13.4 mg g^−1^, 15.7 mg g^−1^, and 39.9 µg g^−1^, respectively. Then SS progressively increased, whereas SP and Pro sharply decreased with the continuous low temperature. Notably, all the SS, SP, and Pro contents returned to the original values before the cold event as temperature recovered (*P* > 0.05). These results above indicated a sequentially synergistic effect of osmolytes, of which SP and Pro worked when initially subjected to the freezing tress, and SS acted during the whole cold event. Generally, exogenous ABA has a promotive effect on the osmotic adjustment substances, among which 100 mg L^−1^ ABA application was the highest.

### Effect of exogenous ABA on enzymatic antioxidants in *K. obovata* during natural frost condition

When initially facing the freezing stress, the activity of superoxide dismutase (SOD, Fig. 1d), peroxidase (POD, Fig. 1e) and catalase (CAT, Fig. 1f) in *K. obovata* leaves dropped dramatically from 624.1 U g^−1^, 1095.1 U g^−1^, and 108.7 U g^−1^ to 540.2 U g^−1^, 456.0 U g^−1^, and 78.4 U g^−1^, respectively (*P* < 0.05). Then POD and CAT gradually increased, and ultimately the activity of CAT was 161.7 U g^−1^, obviously higher than the initial value before the cold event (*P* < 0.05). For SOD, there were no apparent changes with the sustained low temperature (-4.1 ℃ *vs* -5.3 ℃) or restored temperature (9.7 ℃). Meanwhile, the MDA content firstly increased from 23.0 nmol g^−1^ to 34.8 nmol g^−1^, and then kept constant during the cold event (Fig. 1g), suggesting POD and CAT worked together to remove hydrogen peroxide, of which CAT might be more important due to its higher CAT activity. It was evident that exogenous ABA played a positive role in alleviating the oxidative damage to cell membrane triggered by cold stress. For example, the MDA content in *K. obovata* treated with 100 mg L^−1^ ABA reached a maximum of 26.2 ± 2.7 nmol g^−1^ at -5.3 ℃, obviously lower than control check (CK) (36.0 ± 1.1 nmol g^−1^, *P* < 0.05). There was no significant difference among the SOD activity during the cold event after foliar spray of ≥ 50 mg L^−1^ ABA (*P* > 0.05). The SOD activity decreased remarkably in response to cold stress and then returned as temperature recovered when ABA concentration was below 50 mg L^−1^ (*P* < 0.05). Interestingly, the CAT activity firstly decreased to 78.4 ~ 87.2 U g^−1^ and then increased to 156.7 ~ 161.7 U g^−1^, whose variation trend was similar to CK. The decreasing amplitude of POD was mitigated after ABA spraying, and the effect was proportional to the ABA concentration. Finally, the POD activity reached a peak of 991.1 U g^−1^ under ABA 100 mg L^−1^, which was 1.87 times that of CK. To sum up, exogenous ABA effectively improved the cold resistance of *K. obovata* by enhancing the activity of protective enzymes, among which 100 mg L^−1^ ABA was the best one, and no obvious oxidative stress was observed.

### Transcriptome analysis of *K. obovata* under freezing stress and ABA treatment

Based on the physiological changes of *K. obovata*, RNA-Seq was performed on two groups, including 100 mg L^−1^ ABA-treated sample and CK with three biological replicates in each group. After quality control, a total of 1,190,579,508 high-quality clean reads were obtained, and Q20 and Q30 values were both above 97% and 92%, respectively (Table 1). Moreover, the average guanine-cytosine (GC) was more than 45.09%. The de novo assembly using Trinity yielded 33,957 unigenes ranging from 201 to 18,737 bp with an average length of 1,794 bp, and N50 of 3,306 bp (Table 2). The number of unigenes successfully annotated to the NR database was the highest (20,200; 60.62%), followed by COG (18,781; 56.37%), GO (16,867; 50.62%), Swiss-Prot (16,237; 48.73%), Pfam (15,241; 45.74%) and KEGG (9,427; 28.29%) (Supplementary Fig. S2).

**Table 1:** Summary of transcriptome sequencing data and transcriptome assembly

Sample	Clean reads	Clean bases	Error rate (%)	Q20 (%)	Q30 (%)	GC content (%)
CK 10.2 ℃-1	45,719,334	6.82E + 09	0.0265	97.42	92.73	45.09
CK 10.2 ℃-2	50,506,460	7.53E + 09	0.0266	97.38	92.59	45.33
CK 10.2 ℃-3	50,074,114	7.49E + 09	0.0265	97.42	92.68	45.25
CK -4.1 ℃-1	48,129,050	7.19E + 09	0.0266	97.38	92.59	45.4
CK -4.1 ℃-2	53,013,670	7.9E + 09	0.0266	97.42	92.67	45.42
CK -4.1 ℃-3	55,661,348	8.3E + 09	0.0269	97.29	92.39	45.43
CK -5.3 ℃-1	47,262,186	7.06E + 09	0.0271	97.19	92.16	45.4
CK -5.3 ℃-2	52,392,980	7.83E + 09	0.0265	97.43	92.67	45.37
CK -5.3 ℃-3	51,889,784	7.75E + 09	0.0246	98.24	94.5	45.67
CK 9.7 ℃-1	53,295,008	7.95E + 09	0.0266	97.38	92.58	45.19
CK 9.7 ℃-2	49,059,952	7.31E + 09	0.027	97.24	92.29	45.33
CK 9.7 ℃-3	48,207,922	7.17E + 09	0.0266	97.41	92.65	45.34
ABA 10.2 ℃-1	49,330,884	7.37E + 09	0.0274	97.1	91.96	45.25
ABA 10.2 ℃-2	44,899,182	6.7E + 09	0.0263	97.51	92.9	45.33
ABA 10.2 ℃-3	48,488,702	7.22E + 09	0.0264	97.47	92.77	45.39
ABA -4.1 ℃-1	52,589,416	7.84E + 09	0.0262	97.54	92.97	45.18
ABA -4.1 ℃-2	50,639,262	7.54E + 09	0.0262	97.55	93	45.27
ABA -4.1 ℃-3	48,285,670	7.21E + 09	0.0264	97.46	92.79	45.2
ABA -5.3 ℃-1	47,868,554	7.17E + 09	0.0271	97.21	92.21	45.52
ABA -5.3 ℃-2	47,914,928	7.16E + 09	0.0266	97.39	92.62	45.47
ABA -5.3 ℃-3	53,201,112	7.95E + 09	0.0258	97.75	93.28	45.77
ABA 10.2 ℃-1	45,655,372	6.82E + 09	0.0269	97.27	92.33	45.4
ABA 10.2 ℃-2	45,468,018	6.79E + 09	0.0268	97.3	92.43	45.3
ABA 10.2 ℃-3	51,026,600	7.62E + 09	0.025	98.07	94.04	45.82
Total	1,190,579,508

**Table 2:** Statistical results of transcriptome unigenes

Total number	200-500 bp	501-1000 bp	1001-1500 bp	> 1501 bp	N50	Max length	Min length	Average length
33,957	9411	7845	3115	13,586	3306	18,737	201	1793.73

**Fig. 2:**
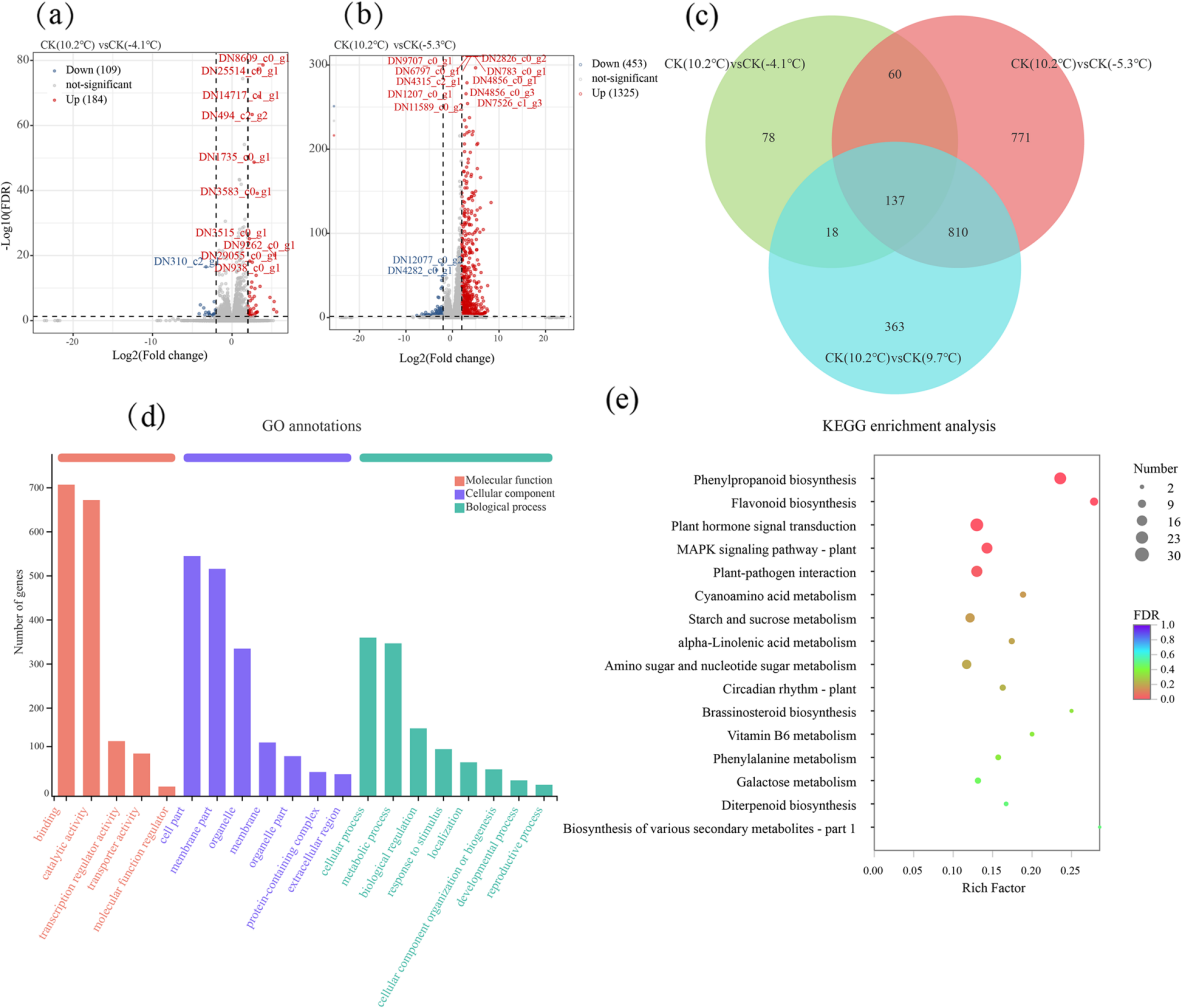
Differentially expressed genes (DEGs) of *K. obovata* during the cold event. **a** Venn diagram, **b** numbers of up and down DEGs, **c** GO annotations, and **d** KEGG enrichment analysis

**Fig. 3:**
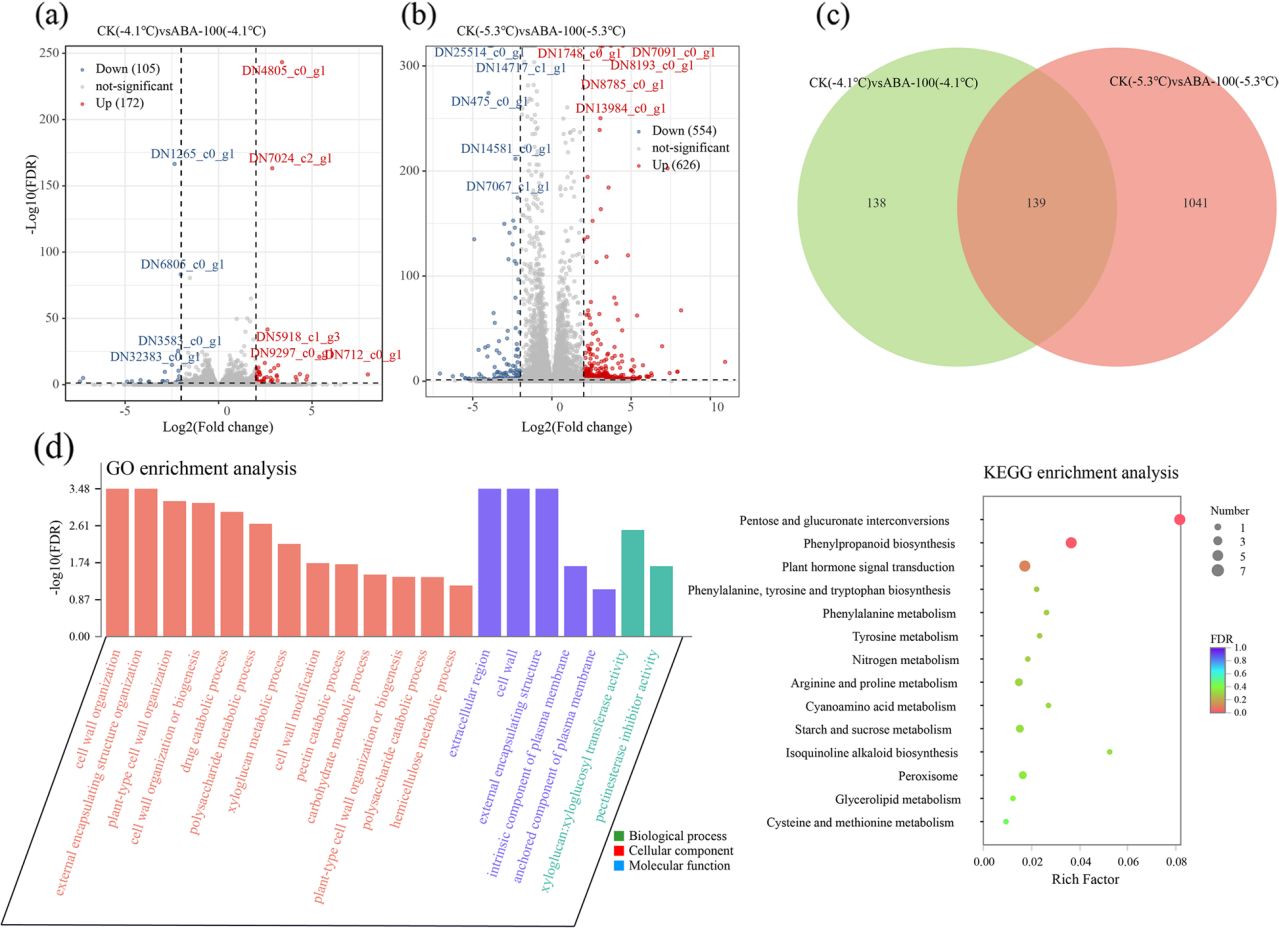
Differentially expressed genes (DEGs) of *K. obovata* between control and ABA group with 100 mg L^−1^ under freezing stress (-4.1 ℃ and -5.3 ℃). **a** and **b** Volcano plot, **c** Venn diagram, **d** GO enrichment analysis, **e** KEGG enrichment analysis

Generally, samples with similar characteristics clustered but samples with different characteristics segregated on score plots. The principal component 1 separated samples from different temperatures during the cold event period, and principal component 2 separated ABA treatment from CK (Supplementary Fig. S3). There were 293, 1,778, and 1,328 differentially expressed genes (DEGs) with annotations in CK (10.2 ℃) *vs* CK (-4.1 ℃), CK (10.2 ℃) *vs* CK (-5.3 ℃), and CK (10.2 ℃) *vs* CK (9.7 ℃), respectively (Fig. 2a). Among them, there were 184, 1,325, and 1,123 up-regulated DEGs, respectively (Fig. 2b). A total of 277 DEGs responding to exogenous ABA were observed under -4.1 ℃, including 172 up-regulated and 105 down-regulated DEGs, respectively (Fig. 3a). Moreover, under -5.3 ℃ there were 626 up-regulated and 554 down-regulated DEGs, respectively (Fig. 3b). To filter preferential ABA-induced cold resistance-related genes, CK (-4.1 ℃) *vs* ABA-100 (-4.1 ℃) and CK (-5.3 ℃) *vs* ABA-100 (-5.3 ℃) was identified using a venn diagram, and 139 DEGs were shared (Fig. 3c).

To clarify the functions of DEGs responding to freezing stress, GO annotations and KEGG enrichment analysis of 1,874 DEGs were conducted (Fig. 2). In biological processes, most DEGs were classified in cellular process and metabolic process, some of which may be associated with responses to stimuli. DEGs in the cellular component and molecular function categories were mainly distributed in cell part, membrane part, organelle, binding, and catalytic activity, respectively (Fig. 2c). A total of 132 KEGG pathways against the cold stress followed in the order: phenylpropanoid biosynthesis, flavonoid biosynthesis, plant hormone signal transduction, MAPK signaling pathway, starch and sucrose metabolism, amino sugar and nucleotide sugar metabolism, phenylalanine metabolism, etc. (Fig. 2d).

**Fig. 4:**
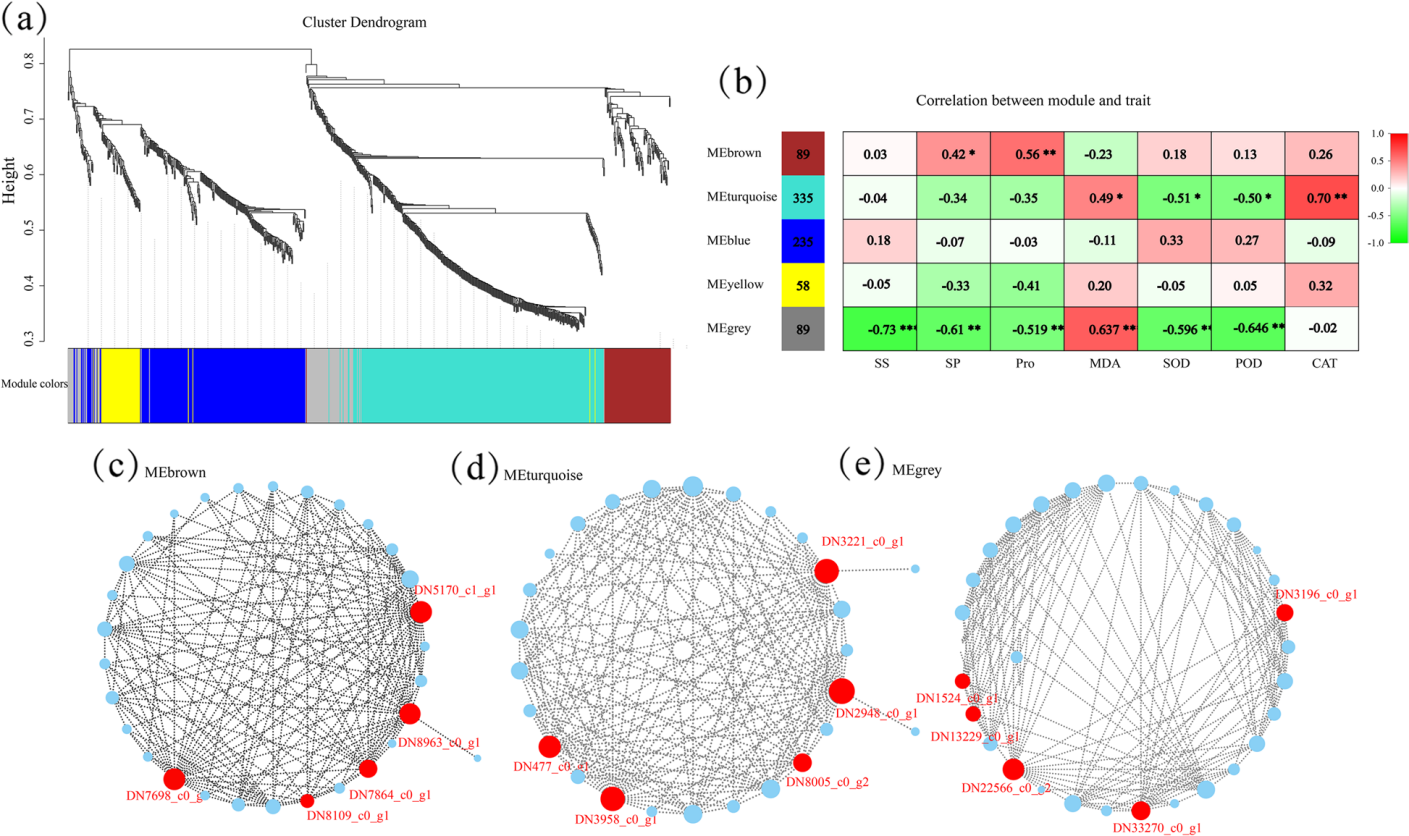
Weighted correlation network analysis (WGCNA) of the candidate genes related to the cold tolerance of *K. obovata*. **A** co-expression modules, **b** heat diagram of correlations between modules and physiological indexes, **c** sub-network of structural genes related to osmotic adjustment substances from brown module, **d** sub-network of structural genes related to antioxidant system from turquoise module, and **e** sub-network of structural genes related to osmolytes and antioxidant system from grey module

To further explore the mechanism of gene response to exogenous ABA, GO enrichment analysis (Fig. 3d) and directed acyclic graph (DAG) (Supplementary Fig. S4) were conducted. In biological processes and cellular component, DEGs were mainly distributed in the cell wall organization (GO:0,009,664) and polysaccharide metabolic process (GO:0,005,976). With respect to molecular function, most of the DEGs were classified in xyloglucan: xyloglucosyl transferase activity (GO:0,016,762). The KEGG result showed these DEGs enriching to pentose and glucoronate interconversions, plant hormone signal transduction, nitrogen metabolism, arginine and proline metabolism, starch and sucrose metabolism, and peroxisome, etc. (Fig. 3e). Additionally, the weighted gene co-expression network analysis (WGCNA) was used to construct a co-expressed gene network module, and a total of 5 modules based on 1,318 unigenes were obtained (Figs. 3c and 4). In the MEbrown module, 89 unigenes had significant positive correlations with SP and Pro (*P* < 0.05), and might play an important role in synthesizing these two osmolytes. The 335 unigenes in the MEturquoise module were significantly related to the SOD, POD, CAT, and MDA (*P* < 0.05), and may be involved in the activities of the enzymatic antioxidants. The 89 unigenes in MEgrey were significantly related to osmolytes and enzymatic antioxidants (*P* < 0.05). Additionally, KEGG pathway enrichments those of 5 hub genes in those three modules were found to be enriched in MAPK signaling pathway-plant, porphyrin and chlorophyll metabolism, sphingolipid metabolism, and phenylpropanoid biosynthesis (Fig. 4 and Supplementary Table S1).

## Discussion

*K. obovata* is an excellent coastal wetland landscape tree in tropical and subtropical shores due to its specific viviparous phenomena, beautiful shape, and unique floral pattern [1]. Although *K. obovata* is the most cold-tolerant mangrove species [9], its northern boundary of artificial cultivation has not yet broken through Yueqing Bay (28°20′ N), Wenzhou [4]. To the best of our knowledge, this is by far the northernmost and lowest temperature field study on mangrove cold resistance. This study provides a foundation for a better understanding of the response in the osmolytes, enzymatic antioxidants, and transcriptome profiling of *K. obovata* under natural frost conditions at ~ 32^o^ N, as well as the acquisition of cold-resistance capability responding to exogenous ABA.

### Temporal variations in physiological status and transcriptome profiling of *K. obovata* under natural frost conditions

Botanical species have evolved several physiological and molecular adaptations, such as osmolytes accumulation, to cope with cold stress [25]. The SS, SP, and Pro are the main compatible solutes, and have an excellent correlation with the cold resistance of *K. obovata* [6, 11–13]. In general, it is known that osmolytes accumulate under cold stress conditions in plants. However, in our study, all the permeable substances, including SS, SP, and Pro, exhibited a remarkably decreased tendency when initially responding to low temperature of -4.1 °C (Fig. 1). Like other subtropical and tropical plants, *K. obovata* is sensitive to low temperature. And previous studies focus mainly on cold stress of *K. obovata* with a temperature ranging from -2 °C to 5 °C [11, 12, 24]. Thus, we speculated that the natural extremely cold event might have serious effects for *K. obovata* and severely lead to short-time metabolism disorder. Fei et al. [12] also observed that the SS of *K. obovata* gradually decreased under chilling stress (5 °C) for 0 h, 1 h, 3 h, and 6 h. Additionally, circadian regulation might be a critically important but neglected factor, and some studies also showed that circadian rhythm is essential for the ability of plants to respond and to acclimate to cold stress, by driving the expression for the (C-repeat-binding factor) *CBF* gene family [28]. Therefore, the SS and Pro gradually increased with progress in persistence of low temperature might be under circadian regulation. Notably, circadian regulation of cold stress is not fully understood, and the role of the circadian rhythm for *K. obovata* at low temperatures requires further investigation. Consistently, transcriptome analysis showed also that the up-regulated DEGs responding to freezing stress were significantly annotated to fructose and mannose metabolism and amino sugar and nucleotide sugar metabolism (Fig. 2d). The SS not only acted as an important osmo-protectant against injuries to the membrane, but also played a cryoprotective role via stabilizing proteins and retaining enzyme activities [29]. Duan et al. [30] reported that accumulation of SS was an important biochemical mechanism to improve freezing tolerance of *Magnolia wufengensis*. Gusta et al. [27] also found sugar had a much greater effect on the freezing process than protein in canola. Therefore, we speculated that both SS and Pro functioned under natural frost conditions, of which SS was more important for *K. obovata*.

Membranes are a primary site of cold-induced injury, whose stability is considered to be a reliable indicator of cellular damage. Low temperature can cause plants to produce a large number of ROS, leading to lipid peroxidation, protein degradation, and enzyme inactivation [25]. The MDA is the final product of membrane peroxidation and reflects the extent of oxidation injure. In the initially stage of cold event, the MDA content of *K. obovata* sharply increased from 23.0 nmol g^−1^ to 34.8 nmol g^−1^ (Fig. 1g). As the first line of the plant's enzymatic defense system, SOD acts as a primary defense against ROS by converting superoxide anion radicals (O_2_-) into molecular oxygen (O_2_) and hydrogen peroxide (H_2_O_2_). Then the POD and CAT mainly play the role of enzymatic degradation of H_2_O_2_ to avoid peroxidation of the cell membrane. Interestingly, the SOD, POD, and CAT activity of *K. obovata* sharply dropped when initially subjected to the freezing tress, which partly attributed to the extreme low temperature (-4.1 °C) causing serious metabolic disorders. Moreover, Jiménez et al. [31] found that most genes involved in ROS scavenging systems are lower expressed during the night. Espinoza et al. [26] observed differences in the cold tolerance of Arabidopsis at the different times (LT50: -8.29 °C in the morning; -7.82 °C in the evening). Thus, the role of the circadian rhythm for *K. obovata* at low temperature, especially the circadian regulation of cold stress and ROS regulation, requires further investigation. As low temperature continued, POD and CAT activity gradually increased, while MDA content kept constant, suggesting POD and CAT work together to remove hydrogen peroxide (Fig. 1e-g). When the cold event passed and temperature rose, only CAT activity was 49% higher than the initial value, suggesting CAT was more important for *K. obovata* to withstand freezing stress under natural frost.

Flavonoid metabolism is an important branch of phenylpropanoid metabolism and gives rise to the largest class of polyphenolic metabolites, approximated to encompass over 8,000 compounds [32]. Flavonoids could also function as antioxidants to inhibit the generation of ROS and reduce ROS levels once formed [33]. Recent studies revealed that cold stress induced the expression of flavonoid structural genes such as *CHS*, *CHI*, *FLS*, and *DFR*, and consequently flavonoids accumulated to facilitate the adaptation to low temperature in *A. thaliana* [34] and *Mikania micrantha* [35]. As shown in Fig. 2d, the top 2 pathways of KEGG enrichment in *K. obovata* were phenylpropanoid biosynthesis and flavonoid biosynthesis, respectively, suggesting that phenylpropanoids, especially the flavonoid, might play vital roles in *K. obovata* coping with natural frost conditions. Thus, further research is needed to investigate the contribution of phenylpropanoid metabolism to cold resistance of *K. obovata*.

### Effect of exogenous ABA on freezing tolerance of *K. obovata* under natural frost condition

Exogenous ABA is being investigated as a novel strategy to improve plants defense against cold stress. Huang et al. [19] found that foliar application of ABA could reduce membrane lipid peroxidation and alleviate cell membrane injury through promoting Pro synthesis in sugarcane seedlings. In anthers, Sharma and Nayyar [15] found that sucrose degradation and transport is regulated by ABA and accumulates in higher amounts under cold stress. Our study showed that exogenous ABA has a promotive effect on the osmolytes, especially for SP and Pro (Fig. 1). Approximately 8,000 chilling-induced genes were observed in *Arabidopsis*, particularly those involved in protein biosynthesis [36]. Gilmour et al. [37] reported that over-expression of *CBF*3 in *Arabidopsis* results in multiple biochemical changes that ultimately increase the concentration of Pro and SS. In the present study, up-regulated DEGs responding to ABA treatment under freezing stress were found to be enriched in nitrogen metabolism, arginine and Pro metabolism, and peroxisome (Fig. 3e). Moreover, we also observed that ABA triggered the expression of *P5CS* and *P5CR*, but inhibited the expression of *ProDH* (Supplementary Fig. S5), which might account for the increase in the Pro content [38]. Pro has also been proposed to function as a molecular chaperone preventing protein aggregation, stabilizing *M4* lactate dehydrogenase [39], protecting nitratereductase [40], and stabilizing ribonucleases and proteases [41] in response to environmental stress. Additionally, Pro accumulation can provide a way to buffer cytosolic pH, balance cell redox status as a ROS scavenger, and store carbon and nitrogen [38]. In chickpea, Kaur et al. [42] reported that the chilling stress injury measured as oxidative stress, electrolyte leakage, loss of chlorophyll and decrease in leaf water content was mitigated significantly after foliar application of Pro. Kumar and Yadav [43] confirmed the protective effects of exogenous Pro to cold stress in *Camellia sinensis* through inhibiting lipid peroxidation as well as by activating or protecting some antioxidants and glyoxalase pathway enzymes. In the bamboo, Liu et al. [44] reported that the alleviation in chilling injury might be caused by enhanced enzyme activities related to Pro metabolism. Therefore, we speculated that Pro accumulation played adaptive roles in *K. obovata* cold hardiness. Improvement of freezing tolerance of *K. obovata* via engineering Pro metabolism is an existing possibility and should be explored more extensively [25]. Notably, the SP and Pro sharply decreased with the duration of low temperature, whereas SS continuously increased, and ultimately the contents of all osmolytes returned to the original values as temperature recovered (*P* > 0.05), implying *K. obovata* did not suffer obvious frost damage after exogenous ABA usage. These results also indicated there might be a sequentially synergistic effect of osmolytes, of which SP and Pro worked immediately facing the freezing stress, and SS acted during the whole cold event.

Exogenous application of ABA could enhance the antioxidant capacity, whose effect might vary positively depending upon the ABA concentration (Fig. 1). Specifically, there were no remarkable changes for SOD and CAT during the cold event under ABA 100 mg L^−1^ (*P* > 0.05), significantly higher than those without ABA spraying initially facing the freezing stress. For POD, the decreasing amplitude was mitigated and the effect was proportional to the ABA concentration. Finally, the POD content was 1.87 times that of CK. These results suggested that exogenous ABA played an active role of radical scavenging performance in *K. obovata* response to cold stress, and more prominently in POD compared with SOD and CAT. Consistently, Rubio et al. [45] also found the combined effect of ABA and low-temperature treatments on the expression of CBF/DREB1 transcription factors VvCBF2, VvCBF3, VvCBF4 and VvCBF6, antioxidant and dehydrin genes, and the acquisition of freezing tolerance. As shown in Fig. 3e, DEGs enriching in peroxisome responding to cold stress after foliar application of ABA, and consequently, there were no significant varieties in MDA contents during the cold event. Those observations resonated with the results of Sandhu et al. [46], and Huang et al. [20], who reported that exogenous ABA triggered the antioxidant defense, and ultimately maintained cell membrane stability and normal function under cold stress.

## Conclusions

To the best of our knowledge, this is by far the northernmost and lowest temperature field study on mangrove cold resistance. This study provides a foundation for a better understanding of the response in the osmolytes, enzymatic antioxidants, and transcriptome profiling of *K. obovata* under natural frost conditions at ~ 32^o^ N, as well as the acquisition of cold-resistance capability responding to exogenous ABA. Specifically, SS played a more important role than Pro in enhancing tolerance to freezing stress. For enzymatic antioxidants, POD and CAT work collaboratively to remove hydrogen peroxide, of which CAT was more important. Transcriptome analysis further indicated that phenylpropanoid metabolism, especially the flavonoid biosynthesis, played a vital role in the cold resistance of *K. obovata*. Exogenous ABA application effectively alleviated the adverse effects of freezing stress on *K. obovata* by increasing the contents of osmotic adjustment substances and enhancing the activities of antioxidant enzyme, especially the Pro and POD. In addition, our findings also offered a sound theoretical foundation for expanding mangroves plantations in higher latitudes, as well as the development coastal landscape.

## Materials and methods

### Plant materials and processing

**Fig. 5:**
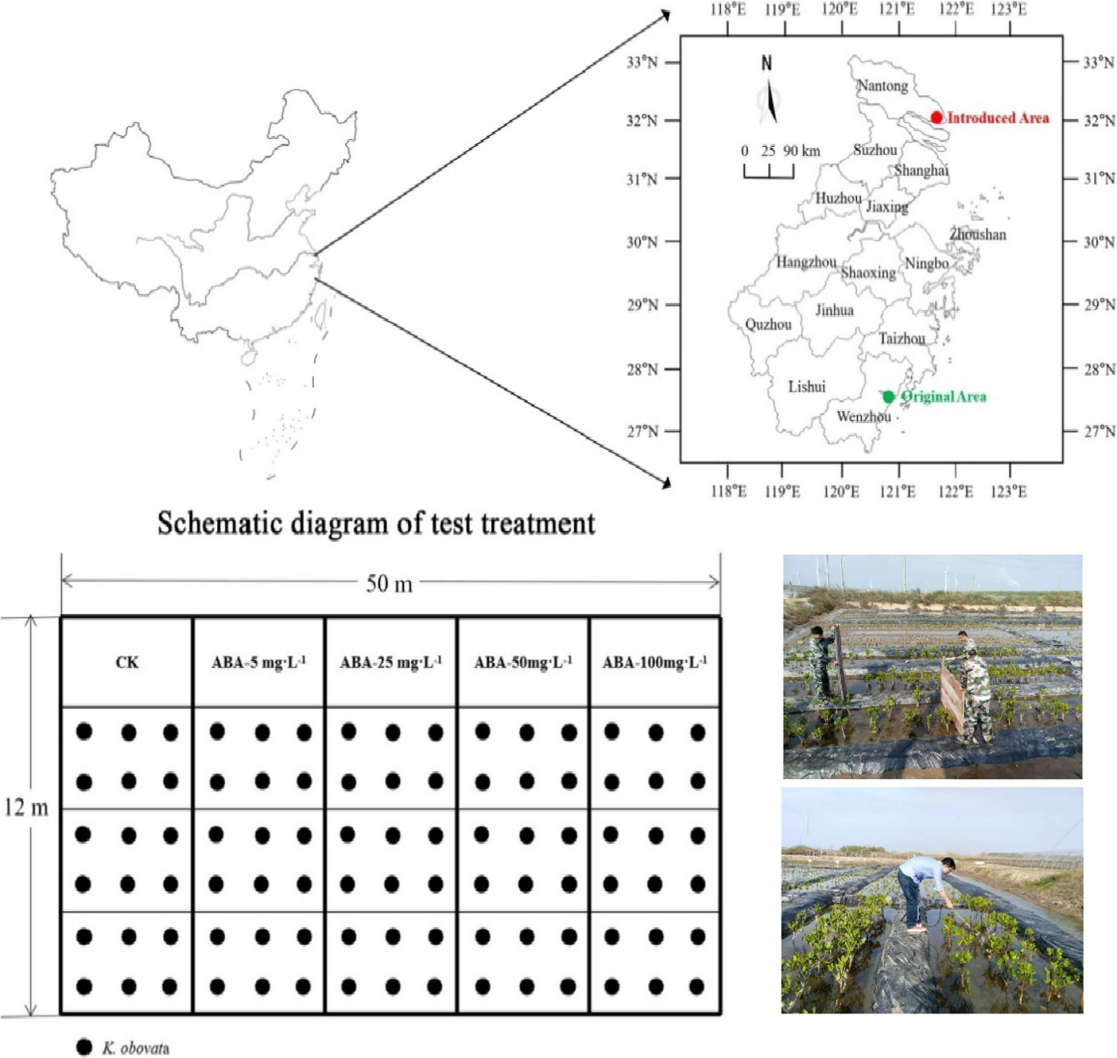
Location of the original area (27^o^56′ N, Shupaisha Island, Wenzhou, Zhejiang Province) and introduced area (31^o^59′ N, Haifu Town, Qidong, Jiangsu Province) of *K. obovata* on the coast of south-eastern China and layout of the experimental treatment

In 2014, we set up seedling garden of *K. obovata* of the Shupaisha wetland, which located in Wenzhou City, Zhejiang Province, China (27°56′ N, 120°51′ E). In early April 2019, ~ 120 three-year-old *K. obovata* seedlings of the similar size were chosen introduced to Qidong (31°59′ N, 121°46′ E), Jiangsu province, and ~ 93% seedlings survived over the summer (Fig. 5). The voucher specimen was deposited in the Zhejiang Institute of Subtropical Crops, Zhejiang Academy of Agricultural Sciences, China. The field planting area was demarcated in fifteen subplots, of which each subplot had 6 seedlings with spacing 0.5 m × 0.5 m.

Cavanaugh et al. [47] identified a temperature-related ecological threshold of -4 °C for mangroves. On December 1–3, 2019, the first frost event (minimum -5.5 °C) occurred at Qidong and gave us a chance to investigate the effects and responses of *K. obovata* when exposed to the natural extremely cold event. At 12:00 a.m. December 1st (10.2 °C), leaves were sampled from each treatment with three subplots for a total of 9 replicate seedlings as CK. ABA (Macklin, China) was dissolved in 98% ethanol (0.1%, v/v) and “Tween-80” was used as the developing agent (0.1%, v/v). In our preliminary experiment, the treatment of ABA 30 mg L^−1^ remarkably improved the cold resistance of *K. obovata*, whose overwintering retention rate increased from 22.6% to 55.0% from 2018 to 2019. Therefore, five different concentrations of ABA (0, 5, 25, 50, and 100 mg L^−1^) were set to treat *K. obovata*. Then at 2:00 p.m. December 1st (11.5 °C) the ABA solution was sprayed with a hand-held sprayer until run off. Finally, leaves were sampled from the same seedling at different stages of the cold event, including 6:00 a.m. on December 2nd (-4.1 °C), 12:00 p.m. on December 2nd (-5.3 °C), 12:00 a.m. on December 3rd (9.7 °C). Then they were immediately washed with distilled water, frozen in liquid nitrogen, and kept at -80℃ until required. Fortunately, there was no obvious morphological damage during this cold event and all seedlings survived in the following spring as it was a warm winter. Consistently, our previous experiment also showed that slight morphological freezing damage occurred in potted eight-month-old seedlings after 12 h subjected to -5.5 °C in the manual climatic box (Fig. S1).

### Physiological and biochemical analyses

The soluble sugar (SS), soluble protein (SP), proline (Pro), superoxide dismutase (SOD), peroxidase (POD), catalase (CAT), and malondialdehyde (MDA) were measured to reveal the varieties of physiological and biochemical status. About 0.1 g of leaf samples were heated in boiling water for 30 min, then centrifuged at 5,000 rpm, and measured SS concentration of the supernatant using the anthrone colorimetric method. For the Pro detection, approximately 0.1 g of leaf samples were homogenized in 3% aqueous sulphosalicylic acid, heated at 100 ℃ for 30 min and filtered. Then the filtrate was mixed with acid-ninhydrin and glacial aceticacid (1:1:1, v/v/v) in a water bath at 100 ℃ for 1 h. Finally, the reaction mixture was extracted with toluene and the absorbance was determined at 520 nm. For the SP, SOD, POD, and CAT detection, approximately 0.1 g of leaf samples was taken into a mortar, and 1 mL ice-cold sodiumphosphate buffer solution (50 mmol L^−1^, pH 7.0) mixed with ethylenediaminetetraacetic acid (1.0 mmol L^−1^) and 2% polyvinylpyrrolidone was added in an ice bath. Then the homogenate was centrifuged at 5,000 rpm, 4℃ for 10 min, and the supernatant was collected. The SP content was measured with the coomassie brilliant blue staining method described by Bradford [48]. The SOD activity was measured following the photoreduction of nitroblue tetrazolium assay [49], while CAT and POD activity were determined according to Wang et al. [6]. For the MDA detection, ~ 0.5 g of leaf samples were placed in 5 mL 10% trichloroacetic acid and centrifuged at 5,000 rpm. Then the supernatant was mixed with 2 mL of 0.67% thiobarbituric acid, heated at 100 ℃ for 30 min, centrifuged at 5,000 rpm, and finally the absorbances at 450, 532, and 600 nm were recorded, respectively. The MDA content was calculated based on the following formula: C (µmol L^−1^) = 6.452 × (A_532_ − A_600_) − 0.559 × A_450_. Each experiment contained three biological and technical replicates.

### RNA extraction, library preparation, and sequencing

A total of 24 independent RNA-Seq libraries from the *K. obovata* leaves of eight groups, with three biological replicates for each group, were constructed and sequenced: CK (10.2 ℃), CK (-4.1 ℃), CK (-5.3 ℃), CK (9.7 ℃), ABA-100 (10.2 ℃), ABA-100 (-4.1 ℃), ABA-100 (-5.3 ℃), and ABA-100 (9.7 ℃). RNA extraction method was provided in the Supplementary data. In addition, reverse transcription, library construction, and sequencing were performed at Shanghai Majorbio Bio-pharm Biotechnology Co., Ltd. (Shanghai, China) and specific operation procedures were provided in the Supplementary data.

### De novo assembly and annotation

The raw paired-end reads were trimmed and quality controlled by SeqPrep (https://github.com/jstjohn/SeqPrep) and Sickle (https://github.com/najoshi/sickle) with default parameters. Then clean data from *K. obovata* samples was used to do De novo assembly with Trinity (http://trinityrnaseq.sourceforge.net/) [23]. All the assembled transcripts were searched against the (National Center for Biotechnology Information) NCBI protein non-redundant (NR), Clusters of Orthologous Genes (COG), and Kyoto Encyclopedia of Genes and Genomes (KEGG) databases using BLASTX to identify the proteins that had the highest sequence similarity with the given transcripts to retrieve their function annotations and a typical cut-off E-values less than 1.0 × 10^–5^ was set. Blast2GO software (http://www.blast2go.com/b2ghome) [50] was used to get gene ontology (GO) annotations for describing biological process, cellular component, and molecular function. Metabolic pathway analysis was performed using the KEGG (http://www.genome.jp/kegg/) [51].

### Differential expression analysis and functional enrichment

The expression level of each transcript was calculated according to the transcripts permillion reads (TPM) method to identify differential expression genes (DEGs). RSEM (http://deweylab.biostat.wisc.edu/rsem/) was used to quantify gene abundance [52]. Essentially, differential expression analysis was performed using the DESeq2 with FDR (*P*-value after adjusting for false discovery rate) ≤ 0.05 and |log2 fold change|> 1 considered to be significant [53]. The Venn diagram was constructed to explore DEGs related to cold tolerance using software available online (http://bioinformatics.psb.ugent.be/webtools/Venn/). The functional-enrichment analysis was performed to identify which DEGs was significantly enriched in GO terms and metabolic pathways at Bonferroni-corrected *P* ≤ 0.05 compared with the whole-transcriptome background. Moreover, GO functional enrichment and KEGG pathway analyses were also carried out by Goatools (https://github.com/tanghaibao/Goatools) and KOBAS (http://kobas.cbi.pku.edu.cn/home.do) [54].

### Statistical analysis

Data are expressed as average ± standard errors of three biological replicates. The statistical analysis was performed by one-way analysis of variance (ANOVA), and Duncan’s multiple range test was employed to separate means at a significant level of *P* < 0.05 were considered significant, using SPSS software version 17.0. The principal component analysis (PCA) was conducted to examine the grouping of samples, outliers, and to visualize the relative distribution of the control and treated samples based on all unigenes identified using Metabo Analyst (https://www.metaboanalyst.ca). After discarding undetectable or relative low expression genes (TPM < 10), weighted gene co-expression network analysis (WGCNA) package in R was used to generate co-expression network modules. The topological overlap-based dissimilarity measure was used to hierarchically cluster all the coding sequences [55]. To make the network show an approximate scale-free topology, using an unsigned type of topological overlap matrix (TOM), the soft threshold power β of six was chosen (model fitting index *R*^2^ > 0.8), a minimal module size of 30, and a branch merge cut height of 0.25. The module eigengene (the first principal component of a given module) value was calculated and used to evaluate the association of modules with SS, SP, Pro, SOD, CAT, POD, and MDA. Moreover, network visualization analysis for modules based on WGCNA was conducted to find the top 5 hub genes.

## Supplementary Information

**Additional file 1: Fig. S1.** Morphologic changes of eight-month-old K. obovata seedlings exposed to freezing stress (-5.5 ℃) in the manual climatic box. **Fig. S2.** Sequences statistics of functional annotation of RNA-Seq data for each database. a venn diagram, b size of each list, c number of tran­scripts or unigenes annotated in databases. **Fig. S3.** Score plots of principal component analysis (PCA) based on all unigenes across control and 100 mg L-1 ABA treated for K. obovata under natural frost conditions. **Fig. S4.** Relationships between GO terms in a directed acyclic graph (DAG). The red to white color represents decreasing significance levels (red is most and white is the least significant). **Fig. S5.** The proline metabolic pathway. Each gene with colored red for up-regulation or blue for down-regulation responding to ABA application under freezing stress (-4.1 ℃ and -5.2 ℃). **Table S1.** GO and KEGG annotations of the top 5 hub gene based on weighted correlation network analysis (WGCNA).

## Data Availability

The RNA-seq raw data in this paper were deposited into the National Center for Biotechnology Information (NCBI) Gene Expression Omnibus database under accession number GEO: GSE219193.
