# Supplementary material for: Role of exogenous abscisic acid in freezing tolerance of mangrove Kandelia obovata under natural frost condition at near 32°N
Source: BMC Plant Biol. 2022 Dec 19;22:593. doi: 10.1186/s12870-022-03990-2 (PMC9762092; doi:10.1186/s12870-022-03990-2)
Supplement: Supplementary file 1 — Additional file 1: Fig. S1. Morphologic changes of eight-month-old K. obovata seedlings exposed to freezing stress (-5.5 ℃) in the manual climatic box. Fig. S2. Sequences statistics of functional annotation of RNA-Seq data for each database. a venn diagram, b size of each list, c number of tran­scripts or unigenes annotated in databases. Fig. S3. Score plots of principal component analysis (PCA) based on all unigenes across control and 100 mg L-1 ABA treated for K. obovata under natural frost conditions. Fig. S4. Relationships between GO terms in a directed acyclic graph (DAG). The red to white color represents decreasing significance levels (red is most and white is the least significant). Fig. S5. The proline metabolic pathway. Each gene with colored red for up-regulation or blue for down-regulation responding to ABA application under freezing stress (-4.1 ℃ and -5.2 ℃). Table S1. GO and KEGG annotations of the top 5 hub gene based on weighted correlation network analysis (WGCNA). [file 12870_2022_3990_MOESM1_ESM.doc]

**RNA extraction, library preparation, and sequencing**

Total RNA from each sample was extracted using TRIzol® Reagent according to the manufacturer’s instructions (Invitrogen, Carlsbard, CA, USA) and genomic DNA was removed using DNase I (TaKara, Shiga, Japan). Then the integrity and purity of the total RNA quality was determined by2100 Bioanalyser (Agilent Technologies, Inc., Santa Clara CA, USA) and quantified using the NanoDrop2000 (Thermo Scientific, Wilmington, DE, USA). Only high-quality RNA sample (OD_260_/OD_280_=1.8~2.2 and OD_260_/OD_230_≥2.0) was used to construct sequencing library.

The RNA-seq transcriptome libraries were prepared using Illumina TruSeq TM RNA Sample Preparation Kit (San Diego, CA, USA). The poly(A) mRNA was purified from total RNA using oligo-dT-attached magnetic beads and then fragmented by using the fragmentation buffer. These short fragments were used as templates and double-stranded cDNA was synthesized using SuperScript Double-stranded cDNA Synthesis Kit (Invitrogen, CA) with random hexamer primers (Illumina). Then the synthesized cDNA was subjected to end-repair, phosphorylation and ‘A’ base addition according to Illumina’s library construction protocol. Libraries were size selected for 200–300 bp cDNA target fragments on 2% Low Range Ultra Agarose followed by PCR amplified usingPhusion DNA polymerase (New England Biolabs, Boston, MA, USA) for 15 PCR cycles. After quantification by TBS380, two RNA-seq libraries were sequenced in a single lane on NovaSeq 6000 sequencer (Illumina, San Diego, CA) for2×150bp paired-end reads.


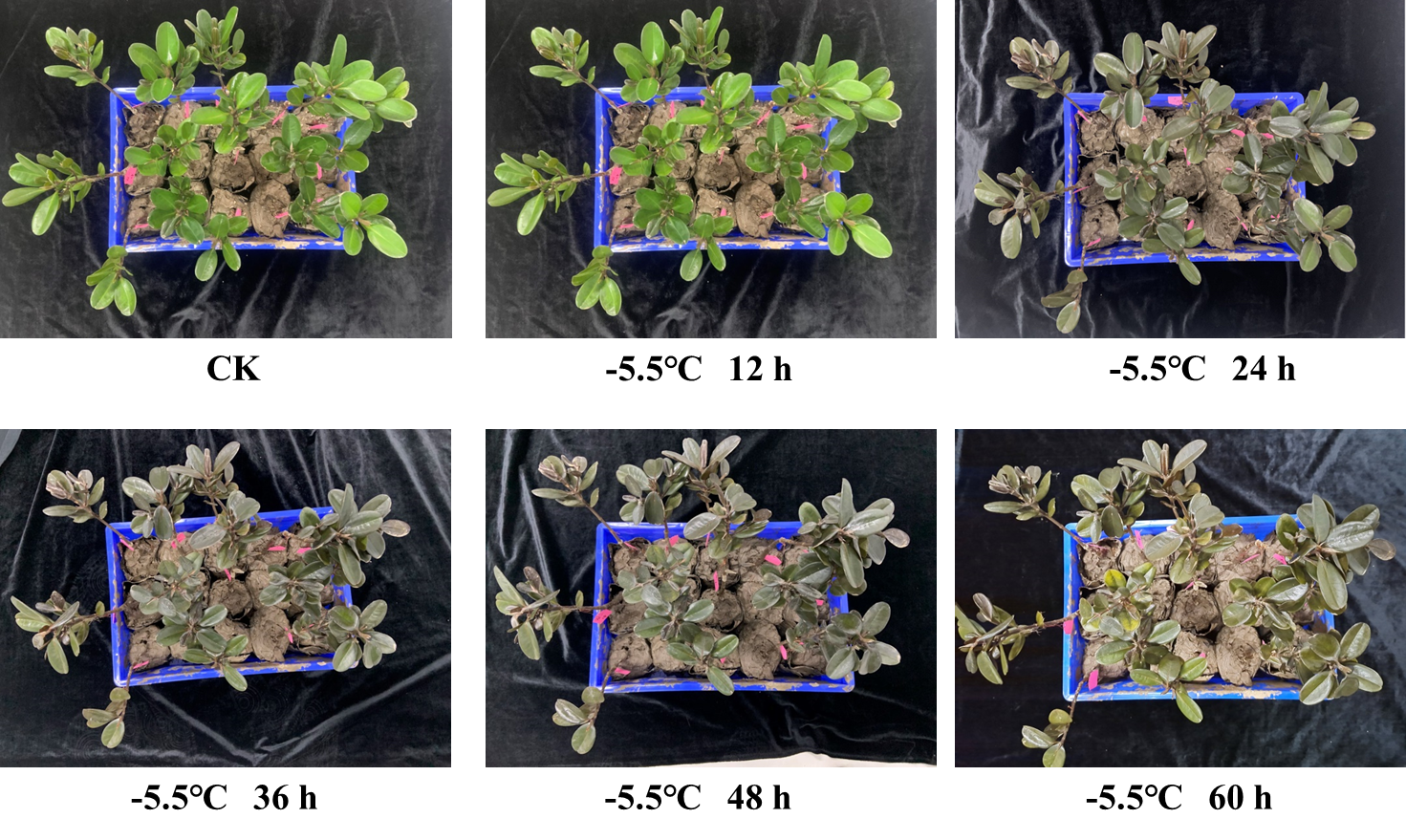


**Fig. S1** Morphologic changes of eight-month-old *K. obovata* seedlings exposed to freezing stress (-5.5 ℃) in the manual climatic box.


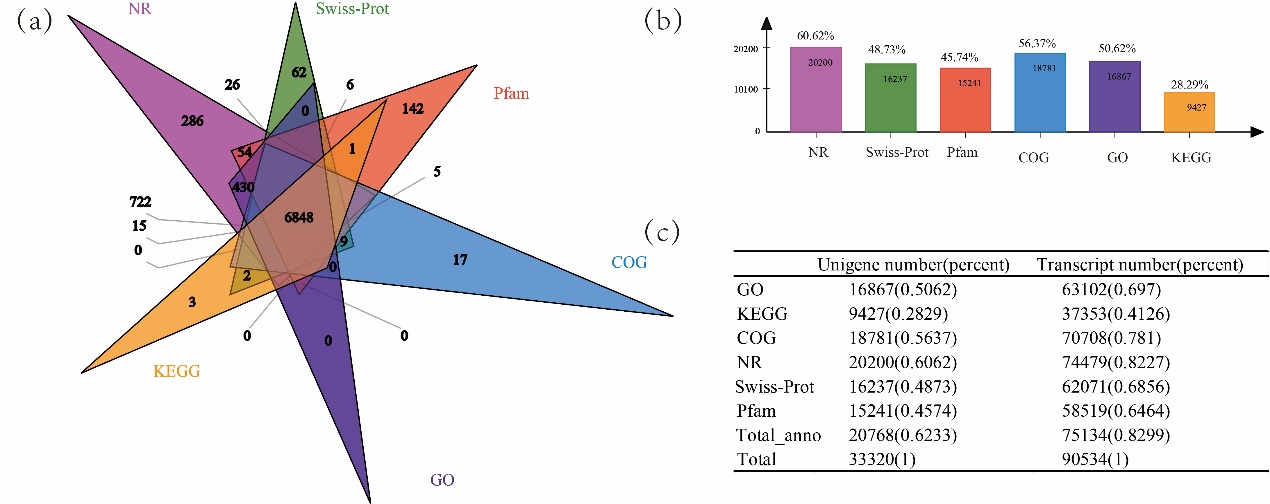


**Fig. S2** Sequences statistics of functional annotation of RNA-Seq data for each database. **a** venn diagram, **b** size of each list, **c** number of tran­scripts or unigenes annotated in databases.


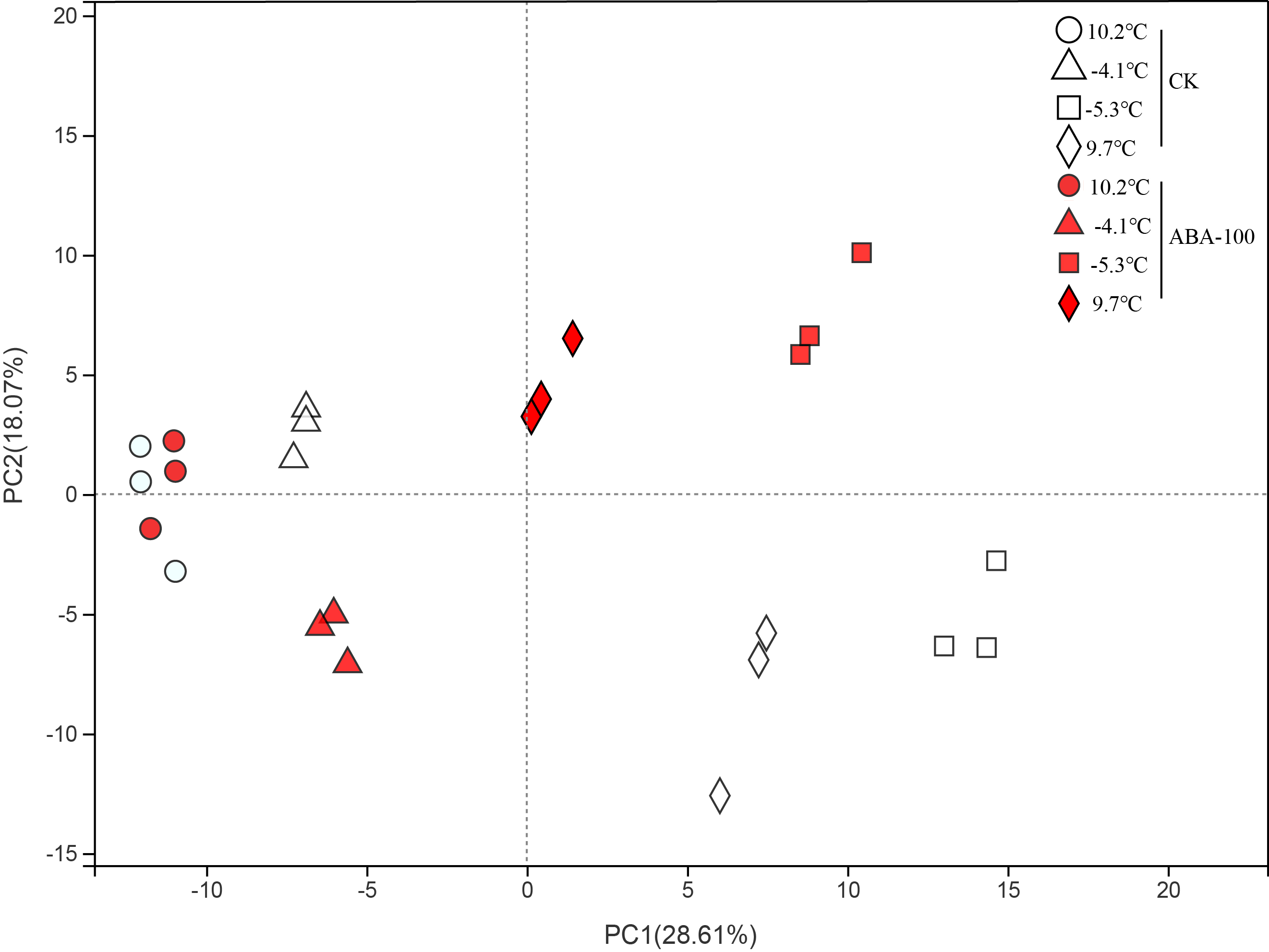


**Fig. S3** Score plots of principal component analysis (PCA) based on all unigenes across control and 100 mg L^-1^ ABA treated for *K. obovata* under natural frost conditions.


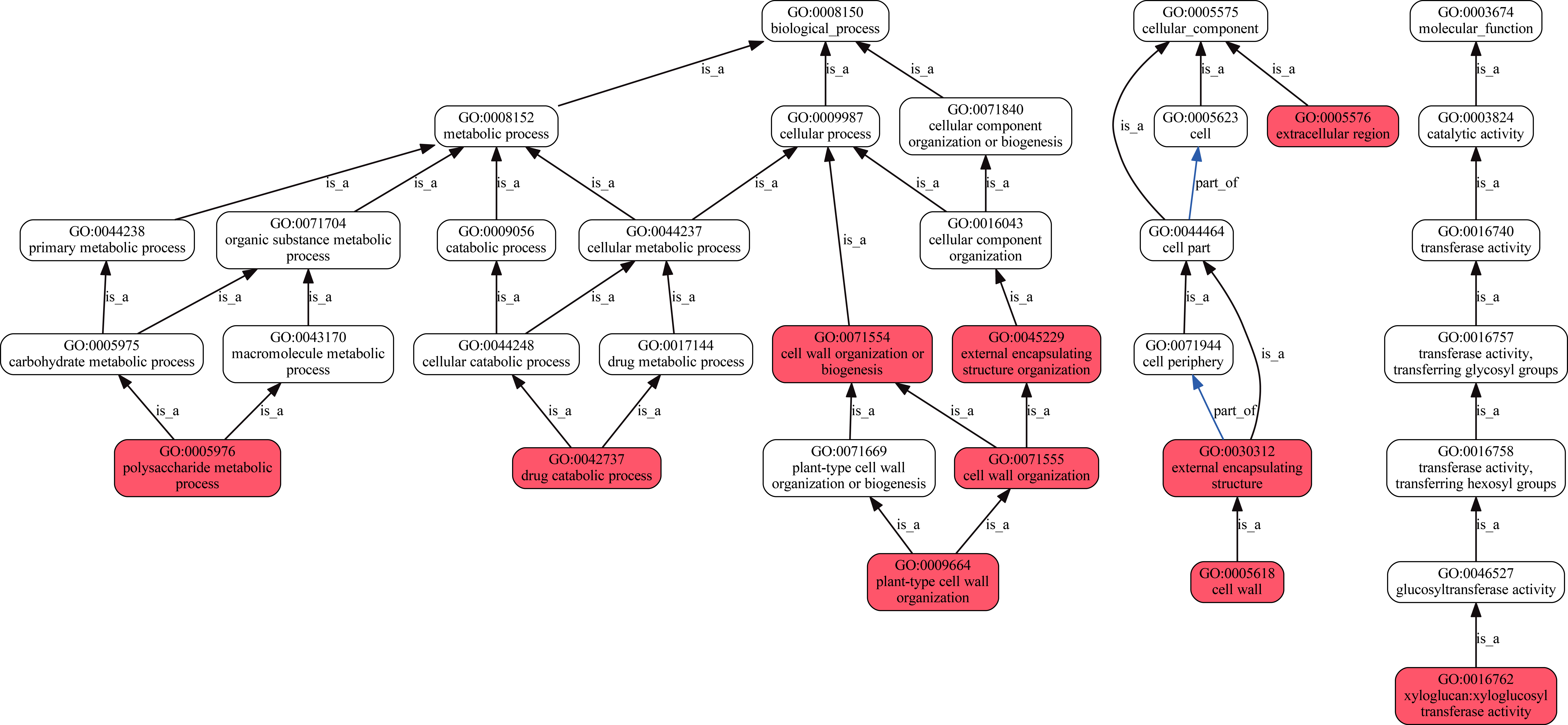


**Fig.** S4 Relationships between GO terms in a directed acyclic graph (DAG). The red to white color represents decreasing significance levels (red is most and white is the least significant).


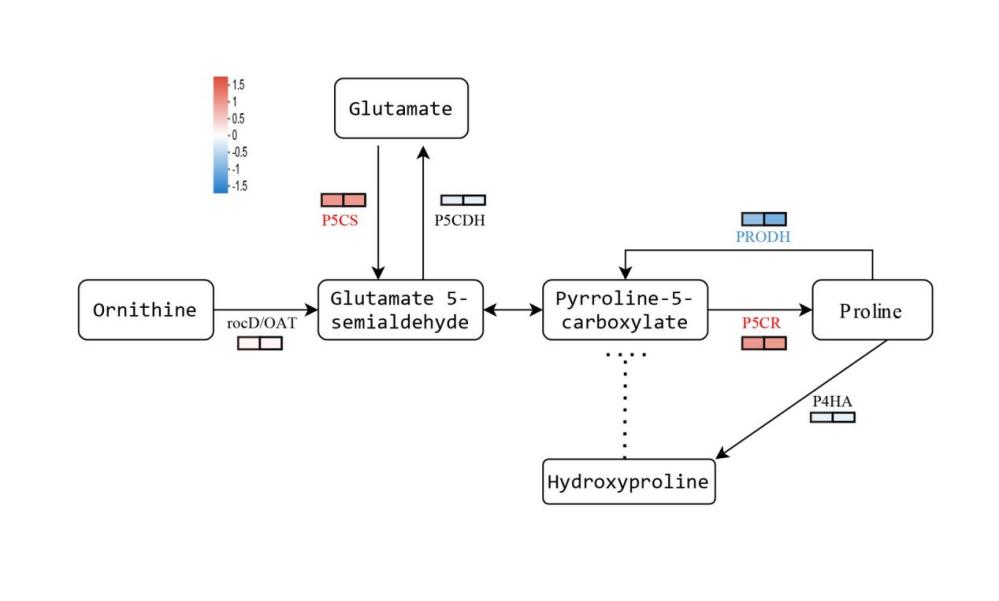


**Fig. S5** The proline metabolic pathway. Each gene with colored red for up-regulation or blue for down-regulation responding to ABA application under freezing stress (-4.1 ℃ and -5.2 ℃).

**Table S1** GO and KEGG annotations of the top 5 hub gene based on weighted correlation network analysis (WGCNA)

| Model | Top 5 gene ID | GO id | GO Description | KO id | Pathway Definition |
| --- | --- | --- | --- | --- | --- |
| MEbrown | DN7698_c0_g1 | GO:0046658 | anchored component of plasma membrane |  |  |
|  | DN5170_c1_g1 | GO:0006355 | regulation of transcription, DNA-templated |  |  |
|  |  | GO:0005634 | nucleus |  |  |
|  |  | GO:0003677 | DNA binding |  |  |
|  | DN8109_c0_g1 | GO:0016021 | integral component of membrane |  |  |
|  | DN7864_c0_g1 | GO:0016021 | integral component of membrane |  |  |
|  |  | GO:0004601 | peroxidase activity |  |  |
|  |  | GO:0005509 | calcium ion binding |  |  |
|  |  | GO:0050664 | oxidoreductase activity, acting on NA | K13447 | MAPK signaling pathway - plant(map04016) |
| MEturquoise | DN2948_c0_g1 | GO:0005634 | nucleus |  |  |
|  | DN3958_c0_g1 | GO:0006783 | heme biosynthetic process | K01772 | Porphyrin and chlorophyll metabolism(map00860 ) |
|  |  | GO:0005739 | mitochondrion |  |  |
|  |  | GO:0016021 | integral component of membrane |  |  |
|  |  | GO:0004325 | ferrochelatase activity |  |  |
|  | DN3221_c0_g1 | GO:0004674 | protein serine/threonine kinase activity |  |  |
|  |  | GO:0005524 | ATP binding |  |  |
|  | DN8005_c0_g2 | GO:0016021 | integral component of membrane | K04710 | Sphingolipid metabolism (map00600 ) |
|  | DN477_c0_g1 | GO:0004722 | protein serine/threonine phosphatase activity |  |  |
|  |  | GO:0004724 | magnesium-dependent protein serine/threonine phosphatase activity | |  |
| MEgrey | DN1524_c0_g1 | GO:0006355 | regulation of transcription, DNA-templated |  |  |
|  |  | GO:0005634 | nucleus |  |  |
|  |  | GO:0008270 | zinc ion binding |  |  |
|  | DN3196_c0_g1 | GO:0016021 | integral component of membrane |  |  |
|  |  | GO:0004672 | protein kinase activity |  |  |
|  |  | GO:0004674 | protein serine/threonine kinase activity |  |  |
|  |  | GO:0004709 | MAP kinase kinase kinase activity |  |  |
|  |  | GO:0005524 | ATP binding |  |  |
|  | DN13229_c0_g1 | GO:0006559 | L-phenylalanine catabolic process | K10775 | Phenylpropanoid biosynthesis (map00940 ) |
|  |  | GO:0009800 | cinnamic acid biosynthetic process |  |  |
|  |  | GO:0005737 | cytoplasm |  |  |
|  |  | GO:0045548 | phenylalanine ammonia-lyase activity |  |  |
